# The Lamellipodin homologue MIG-10 is not essential for dorsal intercalation in the embryonic epidermis of the *C. elegans* embryo

**DOI:** 10.17912/micropub.biology.000522

**Published:** 2022-02-03

**Authors:** Joel M. Serre, Jeff Hardin

**Affiliations:** 1 Program in Genetics, University of Wisconsin-Madison, USA; 2 Department of Integrative Biology, University of Wisconsin-Madison, USA

## Abstract

Dorsal intercalation of the embryonic epidermis in the *Caenorhabditis elegans* embryo is a promising system for genetic analysis of convergent extension, a conserved process in animal embryos. We sought to identify functionally important actin regulators in dorsal epidermal cells. A promising candidate is MIG-10, the single MIG-10/RIAM/Lamellipodin (MRL) family member in *C. elegans*. We endogenously tagged all *mig-10* isoforms with mNeonGreen and analyzed *mig-10* mutants using 4-dimensional microscopy. MIG-10::mNG is expressed prominently in muscle progenitors but is not detectable in the dorsal epidermis. *mig-10(ct41)* homozygotes complete dorsal intercalation in a manner indistinguishable from wildtype, indicating MIG-10 is not essential during dorsal intercalation.

**Figure 1. MIG-10 does not contribute significantly to the process of dorsal intercalation:**
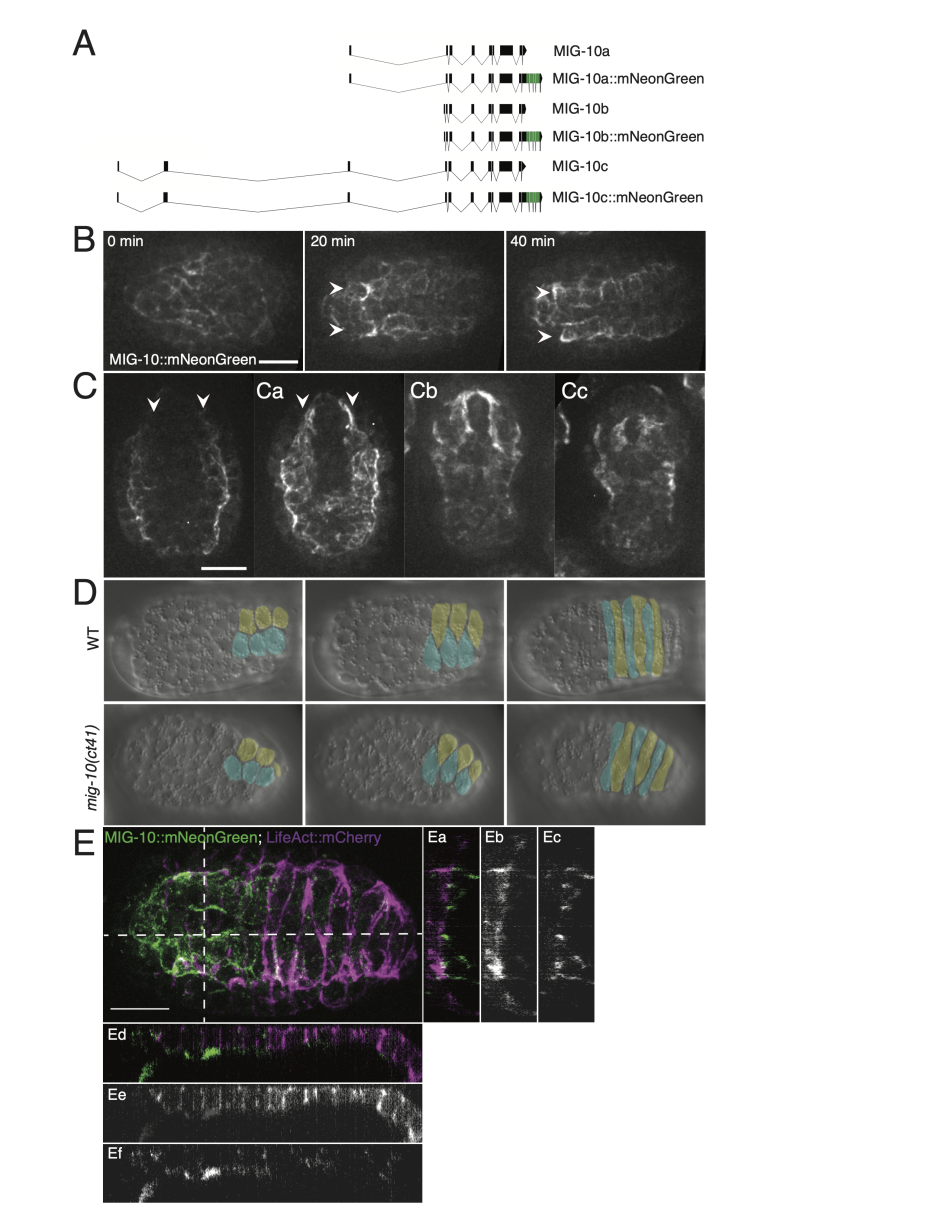
Figure 1. MIG-10 is not essential for the process of dorsal intercalation. (A) Schematic showing the CRISPR-based tagging strategy. All isoforms of MIG-10 include the N-terminal exon to which mNeonGreen (green) was fused. (B-C) MIG-10::mNeonGreen expression shows that MIG-10 isoforms are not appreciably expressed in the dorsal epidermis. (B) MIG-10::mNeonGreen expression from a dorsal view during the process of the dorsal intercalation. (C) MIG-10::mNeonGreen expression from a ventral view before ventral enclosure, (Ca) at the beginning of ventral enclosure, (Cb) during mid ventral enclosure, and (Cc) during early elongation. Arrows indicate muscle quadrants. Images are maximum intensity Z projections of 16 focal planes. Scale bars = 10 µm. (D) DIC images of N2 and *mig-10*(*ct41*) embryos at early, middle and late stages of dorsal intercalation. Posterior cells are pseudocolored for clarity. (E) Confocal image of MIG-10::mNeonGreen with epidermally expressed LifeAct::mCherry. Dotted lines indicate sites of reslicing in Z. Vertical (Ea, Eb, Ec) and horizontal (Ed, Ee, Ef) reslices depicting merged (Ea, Ed), LifeAct::mCherry (Eb, Ee), and MIG-10::mNeonGreen (Ec, Ef) are also shown. Scale bar = 10 µm.

## Description

*Results and Discussion*

Convergent extension, the mediolateral interdigitation of cells to elongate a tissue array along the anterior-posterior axis, is a conserved feature of embryonic development in animals, and underlies such key events in vertebrates as gastrulation and neurulation. In the *Caenorhabditis elegans* embryo a convergent extension-like movement known as dorsal intercalation occurs in the embryonic epidermis. The 20 dorsal epidermal cells are born as two rows of 10 cells each that interdigitate to form a single row that straddles the dorsal midline (Williams-Masson *et al.*, 1998). This process, particularly within the posterior dorsal epidermis derived from the C blastomere, is a highly stereotypical process in wild-type embryos (Williams-Masson *et al.*, 1998), making it a promising system for genetic analysis of a simple example of epithelial convergent extension. Dorsal intercalation requires extension of medially directed basolateral protrusions by intercalating cells regulated by Rho family GTPases, including CED-10/Rac, RhoG/MIG-2, and CDC-42, and downstream mediators, which include the WAVE and WASP regulatory complexes (Walck-Shannon *et al.*, 2016; Walck-Shannon *et al.*, 2015).

We sought to identify additional actin regulators that modulate protrusive activity in dorsal cells. A promising candidate is MIG-10, the single MIG-10/RIAM/Lamellipodin (MRL) family member in *C. elegans*. As the gene name implies, MIG-10 was originally identified in classic genetic screens as a modulator of cell migration (Manser and Wood, 1990). The canonical mutation, *mig-10(ct41)*, results in an amber stop in Exon 3, resulting in predicted severe truncation and strong loss of function of both MIG-10 isoforms (Manser *et al.*, 1997). In neurons, MIG-10 binds activated CED-10/Rac, which polarizes it to the leading edge of growth cones and is necessary for proper axon guidance (Quinn *et al.*, 2008).

Given established requirements for MIG-10 in cell movement and its connection to Rac signaling, we investigated whether MIG-10 plays a significant role during dorsal intercalation in two ways: (1) by endogenously tagging all *mig-10* isoforms with mNeonGreen, and (2) by analyzing *mig-10* mutants using 4-dimensional DIC microscopy. We endogenously tagged MIG-10 using a C-terminal insertion in the last exon, which is shared by all *mig-10* isoforms (Fig. 1A). The endogenous insertion maintains full function of MIG-10, since we did not observe any Mig phenotypes in embryos, larvae, and adults homozygous for the endogenously tagged version of the gene. The earliest prominent expression of MIG-10 we observed was during early morphogenesis in ventral neuroblasts and muscle precursors. The latter organize into four quadrants, two dorsal and two ventral, as embryogenesis proceeds (Viveiros *et al.*, 2011). The two dorsal muscle quadrants lie immediately ventral to the dorsal epidermis, and show prominent expression of MIG-10::mNG (Fig. 1B, 1C). While it is possible that there is low-level expression of *mig-10* in dorsal epidermal cells, if it is expressed there it is present at much lower levels than in dorsal muscle quadrants. We did not see any overlap between epidermally driven LifeAct::mCherry and MIG-10::mNeonGreen (Fig. 1E), further suggesting that detectable dorsal *mig-10* expression is restricted to muscle precursors at this stage of development.

The relative lack of expression of MIG-10 in the dorsal epidermis suggests that it is not a major contributor to actin dynamics during dorsal intercalation. We confirmed this using 4d DIC microscopy. *mig-10(ct41)* homozygotes complete dorsal intercalation in a manner indistinguishable from wild-type embryos (Fig. 1D). In 8 mounts with 21 dorsal presenting embryos, we observed no dorsal intercalation defects in the *mig-10*(*ct41*) background, a result indistinguishable from wild-type embryos (no defects in 7 mounts with 26 dorsal presenting wild-type embryos).

Taken together, these results suggest that MIG-10 is not an essential actin regulator in dorsal epidermal cells during dorsal intercalation. It will be interesting to determine if MIG-10 acts redundantly with other actin regulators or if it plays more prominent roles during other cell migration events in the embryo.

## Methods

*Strains*

Hermaphrodite worms were maintained at 20°C on NGM plates with OP50. Strains used: Bristol (N2), SU875 *mig-10*(*jc53*[*mig-10::mNeonGreen::3xFlag* + *LoxP*]III, SU876 (derived from strain BW315, *mig-10(ct41)* 3x outcrossed into N2). SU1052 *mig-10*(*jc53*[*mig-10::mNeonGreen::3xFlag* + *LoxP*]III; *curIs11*[*Plin-26::LifeAct::mCherry::unc-54 3’UTR; unc-119 (+)*].

*Confocal imaging*

MIG-10::mNeonGreen embryos were dissected from adult hermaphrodites and mounted onto 10% agar pads in M9 solution and imaged essentially as described (Zaidel-Bar *et al.*, 2010). Spinning-disc confocal images were acquired with a Z-slice spacing of 0.5 μm using µManager software v1.4.18 (Edelstein *et al.*, 2014) using a Nikon Eclipse E600 microscope controlled via a Prior Z motor (Prior Scientific Instruments, Rockland, Massachusetts) connected to a Yokogawa CSU10 spinning disk scanhead (originally purchased from Perkin-Elmer Life Sciences), a Vortran solid state laser launch controlled using an Arduino control interface (purchased from BioVision Technologies, Exton, Pennsylvania), and a Hamamatsu ORCA-ER charge-coupled device (CCD) camera (Hamamastu Photonics USA, Bridgewater, New Jersey).

*4d DIC analysis*

Embryos were dissected from adult hermaphrodites and mounted onto 10% agar pads in M9 solution and imaged as described (Walck-Shannon *et al.*, 2015). Image stacks were obtained at 90s intervals. Embryos were scored for dorsal intercalation defects (ipsilateral comigration and failure to intercalate) among the posterior cells of the dorsal array. Color overlays were produced in ImageJ as described (Walck-Shannon *et al.*, 2015).

*Genomic Diagrams*

Exon-intron diagrams were made using the Exon-Intron Graphic Maker designed and hosted by Nikhil Bhatla. This tool can be accessed at wormweb.org/exonintron

*CRISPR*

To generate the MIG-10::mNeonGreen knock-in line, we used a plasmid-based self-excising cassette methodology that has been described previously (Dickinson *et al.*, 2015). To generate the appropriate repair template, *pJMS5*, Gibson cloning was used to replace ccdB sequences in *pDD268* with a 5’ homology arm that was approximately 433bp upstream of the Stop codon and a 3’ homology arm beginning with the stop codon and continuing downstream for approximately 538bp amplified from single-worm lysates. The homology arms flanked a self-excising cassette (SEC) which contains a dominant rol allele of *sqt-1,* a heat shock inducible *Cre* recombinase and a hygromycin drug resistance marker*.* *pDD268* was generously provided by Dan Dickinson. The guide sequence was cloned into *pJW1219* to make *pJMS4*. *pJW1219* was a gift from Jordan Ward (Addgene plasmid # 61250 ; http://n2t.net/addgene:61250 ; RRID:Addgene_61250) (Ward, 2015). *pJMS5* was injected at 15 ng/ml into N2 worms with *pJMS4* (50ng/ml; *pJW1219* carrying the guide sequence for the C-terminal end of *mig-10* and *Cas9)* and co-injection markers *pGH8 (Prab-3::mCherry*, 10 ng/ml), *pCFJ104* (5 ng/ml*, Pmyo-3::mCherry*),and *pCFJ90 (Pmyo-2::mCherry*, 2.5 ng/ml).Following selection for rolling worms that no longer carried the extra-chromosomal array, we selected for homozygous rolling worms. These worms were then heat shocked at 32°C for 4 hours to remove the SEC, which left a *LoxP* site in a synthetic intron between the *mNeonGreen* and *3xFlag* tags*.*

Sequences for homology arm amplification primers and the sgRNA were as follows:

5’ homology arm Forward primer- 5’-gccaacaatctcatccatctcg-3’

5’ homology arm reverse primer- 5’-acactccatggttgccattttctc-3’

3’ homology arm forward primer-5’-tagacaacatttagaatactggata-3’

3’ homology arm reverse primer-5’-tgtatgcaactgtggaatat-3’

sgRNA 5’-aaatgttgtctaacactcca**–**3’
